# High-Fructose Diet Increases Renal *ChREBP*β Expression, Leading to Intrarenal Fat Accumulation in a Rat Model with Metabolic Syndrome

**DOI:** 10.3390/biology11040618

**Published:** 2022-04-18

**Authors:** Ariel Bier, Eliyahu Shapira, Rawan Khasbab, Yehonatan Sharabi, Ehud Grossman, Avshalom Leibowitz

**Affiliations:** 1 Medicine D, The Chaim Sheba Medical Center, Tel-Hashomer, Ramat Gan 5262000, Israel; arielbier@gmail.com (A.B.); eli.shapira92@gmail.com (E.S.); yehonatan.sharabi@sheba.health.gov.il (Y.S.); ehud.grossman@sheba.halth.gov.il (E.G.); 2 Sackler Faculty of Medicine, Tel-Aviv University, Tel Aviv 6997801, Israel; 3 Hypertension Unit, The Chaim Sheba Medical Center, Tel-Hashomer, Ramat Gan 5262000, Israel; rawankhasbab@gmail.com

**Keywords:** fructose, metabolic syndrome, fatty kidney, *ChREBP*β

## Abstract

**Simple Summary:**

Fructose consumption leads to the development of metabolic syndrome. Fatty liver and chronic kidney disease are closely related to metabolic syndrome. Lately, a transcription factor that regulates fructose metabolism in the liver, named *ChREBP*β, which is responsible for de-novo lipogenesis and intra-hepatic fat accumulation (“fatty liver”), was described. In this study, we demonstrate that the effect of fructose consumption on the kidneys resembles its liver effect. Rats fed with a high-fructose diet exhibit bigger kidneys with higher triglycerides content, compared to control rats. The expression of *ChREBP*β and its downstream genes was upregulated as well. Treating kidney-origin cells with fructose increased the expression of this factor as well, showing the direct effect of fructose on this factor. Thus, the appearance of fatty kidney in response to high-fructose consumption revealed a new mechanism linking metabolic syndrome to chronic kidney disease.

**Abstract:**

Fructose consumption is associated with metabolic syndrome (MeS). Dysregulated lipid metabolism and ectopic lipid accumulation, such as in “fatty liver’’, are pivotal components of the syndrome. MeS is also associated with chronic kidney disease (CKD). The aim of this study was to evaluate kidney fructose metabolism and whether the addition of fructose leads to intrarenal fat accumulation. Sprague Dawley rats were fed either normal chow (Ctrl) or a high-fructose diet (HFrD). MeS features such as blood pressure and metabolic parameters in blood were measured. The kidneys were harvested for *ChREBP*β and de novo lipogenesis (DNL) gene expression, triglyceride content and histopathology staining. HK2 (human kidney) cells were treated with fructose for 48 h and gene expression for *ChREBP*β and DNL were determined. The HFrD rats exhibited higher blood pressure, glucose and triglyceride levels. The kidney weight of the HFrD rats was significantly higher than Ctrl rats. The difference can be explained by the higher triglyceride content in the HFrD kidneys. Oil red staining revealed lipid droplet formation in the HFrD kidneys, which was also supported by increased adipophilin mRNA expression. For *ChREBP*β and its downstream genes, scd and fasn, mRNA expression was elevated in the HFrD kidneys. Treating HK2 cells with 40 mM fructose increased the expression of *ChREBP*β. This study demonstrates that fructose consumption leads to intrarenal lipid accumulation and to the formation of a “fatty kidney”. This suggests a potential mechanism that can at least partially explain CKD development in fructose-induced MeS.

## 1. Introduction

Obesity, impaired glucose metabolism, dyslipidemia and hypertension are the classical features of metabolic syndrome (MeS). Despite not being included in the formal definitions, MeS is frequently associated with fatty liver and chronic kidney disease (CKD) [1], and CKD is also connected to non-alcoholic steatohepatitis (NASH) and insulin resistance (IR) [2,3]. Mechanistically, the secretion of pro-inflammatory adipocytokines, uric acid toxicity, mitochondrial dysfunction and oxidative stress have been all suggested as possible mechanisms that contribute to MeS-related CKD.

Ectopic fat accumulation, mainly in the liver and muscle tissue, is a well-established pathology in MeS. Emerging evidence suggests that ectopic fat accumulation is also a pathological process occurring in the kidney (reviewed in [4]). However, the linkage to MeS is not clear.

Data from both adult and child autopsies show a correlation between body mass index (BMI) and kidney weight [5]. In obese individuals, there is an enlargement of several organs such as the liver, heart and kidneys [6]. Data from animal models of obesity and MeS, including the high-fructose diet (HFrD) model, support this notion as well [7]. Kidney enlargement was attributed to glomerular and tubular hypertrophy, hemodynamic changes and fluid retention [8]. Lately, the possibility of altered lipid metabolism and intra-renal fat accumulation has been suggested, with most of the evidence coming from studies of obesity and high-fat diet models [9,10,11,12,13].

The Western diet plays a major role in the increased prevalence of MeS and obesity, and epidemiological studies show that the most significant Western diet component that has increased tremendously in last decades is fructose [14]. High-fructose consumption leads to MeS, NASH and IR, and therefore probably also contributes to the development of CKD. However, despite fructose’s wide use, the data regarding kidney fructose metabolism is scarce. Most of the studies have focused on the kidney’s ability to metabolize fructose to glucose since the kidneys are key players in regulating blood glucose levels [15,16]. A few studies have started to uncover that ectopic fat accumulation in the kidneys follows a HFrD [17,18]. However, still no convincing evidence or mechanisms have been presented for HFrD intrarenal fat accumulation.

The transcription factor carbohydrate responsive element binding protein (*ChREBP*) is a regulator of lipogenesis and gluconeogenesis [19]. Herman et al. recently described a new potent *ChREBP* isoform, *ChREBP*β [20]. High expression levels of hepatic *ChREBP*β are associated with insulin resistance [21]. Recently, fructose metabolism in the liver was found to be mainly regulated by *ChREBP*β. It was also demonstrated in this study that ChREBPβ is responsible for fructose-induced “fatty liver” and liver insulin resistance [22]. We therefore hypothesized that HFrD induces “fatty kidney” in the same manner that it induces “fatty liver”.

The aim of this study was to reveal whether kidney fructose metabolism is associated with *ChREBP*β upregulation and whether its metabolism leads to lipogenesis and intrarenal lipid accumulation.

## 2. Materials and Methods

### 2.1. Animals and Study Design

Sprague–Dawley male rats, weighing 200 ± 20 g (Harlan Laboratories, Jerusalem, Israel), were housed in regular cages at 22 °C using a 14-h light (6:00–20:00)/10-h dark (20:00–6:00) cycle. The rats had free access to food and water. Study procedures were in accordance with the Chaim Sheba Medical Center’s guidelines for animal studies and were approved by our institutional animal ethics committee. Rats were fed either a control rodent diet (*n* = 7) or a high-fructose (60%) diet (*n* = 8), for eight weeks. To prove the development of MeS, body weight, blood pressure (BP), fasting blood glucose and triglycerides were measured at the end of the treatment period. After sacrificing, the kidneys were weighed and sliced for histology studies (oil red staining of frozen sections). The kidney tissue was immediately snap-frozen in liquid nitrogen and stored at −80 °C. Frozen tissues were used for RNA extraction and for measuring total tri-glycerides.

### 2.2. Diets

The HFrD (TD.89247 Teklad Envigo Madison, WI, USA) consisted of 60% fructose. The normal chow control diet (TD.170308; Teklad Envigo Madison, WI, USA) was a custom diet matching the HFrD. A detailed comparison of the diets is provided in Table 1.

### 2.3. Metabolic Syndrome Features

Glucose and Tg levels were measured with an automated analyzer for an enzymatic colorimetric reaction (Olympus AU 2700, Hamburg, Germany).

Systolic BP was measured by the indirect tail cuff method, using an electrosphygmomanometer and pneumatic pulse transducer (58500 BP Recorder, UGO BASILE, Varese, Italy). We used the mean of five consecutive readings as the BP value.

For measuring total kidney triglycerides, one kidney was freeze dried by a lyophilizer and then mashed using a mortar and pestle. The total kidney was taken for triglyceride measurement using a triglyceride colorimetric assay kit (Cayman #10010303, Cayman chemical, Ann Arbor, MI, USA).

### 2.4. Real-Time Quantitative PCR

mRNA expression levels were ascertained in the kidney tissue by real-time quantitative reverse transcriptase (RT) PCR (qRT-PCR). Total RNA was extracted from the kidney tissue by the NucleoSpin RNA Kit (Macherey-Nagel, Düren, Germany). Reverse transcription was performed using the Applied Biosystems High Capacity cDNA Reverse Transcription Kit (Applied Biosystems, Foster City, CA, USA). qRT-PCR reactions were performed by the Power Sybr Green PCR Master Mix (Applied Biosystems, Warrington, UK) using the Applied Biosystems 7500 Real-Time PCR System. Ribosomal protein lateral stalk subunit P0 (Rplp0) mRNA was used as an internal control. The primers are listed in Table 2.

### 2.5. Oil Red O Staining (ORO)

ORO staining was done on frozen kidneys that were sliced by a microtome to 10-μm-thick sections. The sections were dried for 10 min at RT before being frozen, and then they were stored at −80 °C. ORO was performed by making stock and working solutions. Stock solution: 2.5 g of ORO (Sigma o0625-25g) to 400 mL of 99% (vol/vol) isopropyl alcohol and mixing by a magnetic stirrer overnight at room temperature (RT; 20–25 °C). Working solution: 1.5 parts of ORO stock solution to one part of distilled water. Incubation was for 5–10 min at 4 °C, during which time the solution thickened. Filtration through a 45-μm filter was carried out to remove precipitates. The working solution was used within 6 h. Sections were submerged in a slide holder with ORO working solution at RT for 5 min. Sections were then rinsed under running tap water for 30 min. The sections were counterstained with Mayer′s hematoxylin by submerging the sections in hematoxylin for 15 s and thereafter submerging them in water for 5 min. The slides were mounted with Shandon ImmuMount (Thermo scientific), and after 10 min at RT, coverslips were placed on them and the coverslip edges were sealed with nail polish. This protocol was based on the protocol of Mehlem et al. [24]. Images were captured by an Olympus BX43 microscope equipped with a digital camera (Olympus DP71) using the software Entry CellSens Ver.2.1. (Olympus Europa SE and CO. KG, Hamburg, Germany).

### 2.6. Cell Culture

The kidney, cortex/proximal tubule human cell line, and HK2 (ATCC^®^ CRL-2190™) were obtained from ATCC^®^. Cells were cultured in DMEM high glucose (01-055-1A, biological industries, Beit-Haemek, Israel) with 1% L-glutamine, 1% pen-strep and 10% fetal bovine serum. When cells were treated with fructose or glucose, the cells were cultured in DMEM no glucose (01-057-1A, biological industries, Beit-Haemek, Israel) with 1% L-glutamine, 1% pen-strep and 10% fetal bovine serum and fructose or glucose were added. The cells were incubated with fructose or glucose for 48 h. All experiments were done in six well plates, 2.5 × 10^5^ cells per well.

### 2.7. Statistical Analysis

Data are presented as mean ± S.E.M. For statistical calculations, we used the IBM^®^ SPSS^®^ Statistics, version 24 (IBM Corporation, Armonk, NY, USA). Differences between two groups were obtained by an unpaired *t-*test. For RT qPCR data analysis, we used DataAssist software, version v3.01 (Applied Biosystems, Life Technologies Corporation 2012, now under Thermo Fisher Scientific, Waltham, MA, USA). A *p* value of ≤0.05 was considered statistically significant. Excel^®^ and Graph Pad Prism8^®^ were used to create the graphs.

## 3. Results

### 3.1. The Effects of HFrD on Metabolic Featured

**Figure 1:**
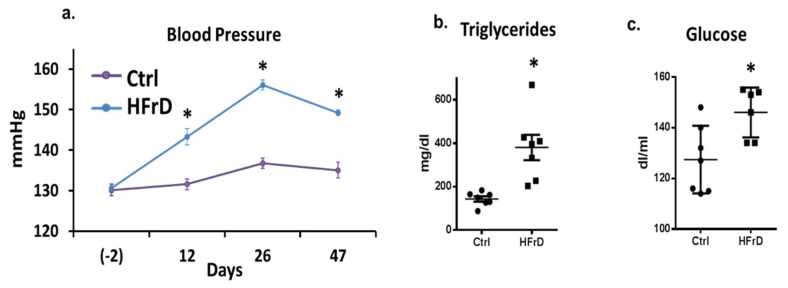
High-fructose diet (HFrD)-fed rats developed hypertension and increased blood triglycerides and glucose level. Blood pressure (BP) was measured using tail cuff (**a**). Blood triglycerides and glucose were measured at the end of the experiment (**b**,**c**). * *p* ≤ 0.05. *n* = 7–8.

As expected, HFrD induced MeS in the rats. BP, fasting blood triglycerides and glucose levels were significantly higher in HFrD-fed rats in comparison to control rats (Figure 1a–c).

### 3.2. HFrD Is Associated with Ectopic Lipid Accumulation in the Kidney

**Figure 2:**
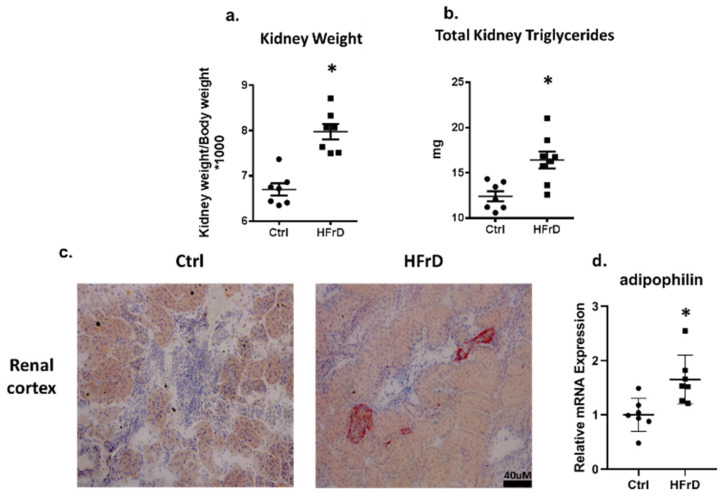
HFrD increased kidney weight and induced ectopic lipid accumulation in the kidneys. Kidney weight was measured at the end of the study and was normalized to body weight (**a**). Total kidney triglycerides were measured using a colorimetric Assay Kit (**b**). Ectopic lipid accumulation was measured by oil red O staining in the renal cortex, representative slides, and 20× magnification (**c**). Kidney adipophilin expression levels were measured by real time PCR (**d**). * *p* ≤ 0.05. *n* = 7–8.

HFrD-fed rats exhibited heavier kidneys in comparison to control rats (Figure 2a). Total kidney triglycerides were measured by a colorimetric assay kit, and significantly higher amounts were detected in the HFrD-rats, which can at least partially explain their higher weight (Figure 2b). Ectopic lipid accumulation was detected in the HFrD-fed rat kidneys by oil red staining for lipid droplets in the cortex (Figure 2c). Moreover, the expression of adipophilin, a key gene in the structural component of the lipid droplet, was increased in this group (Figure 2d).

### 3.3. HFrD Increases Kidney ChREBPβ Expression and Other Genes Involved in Carbohydrate and Fat Metabolism

**Figure 3:**
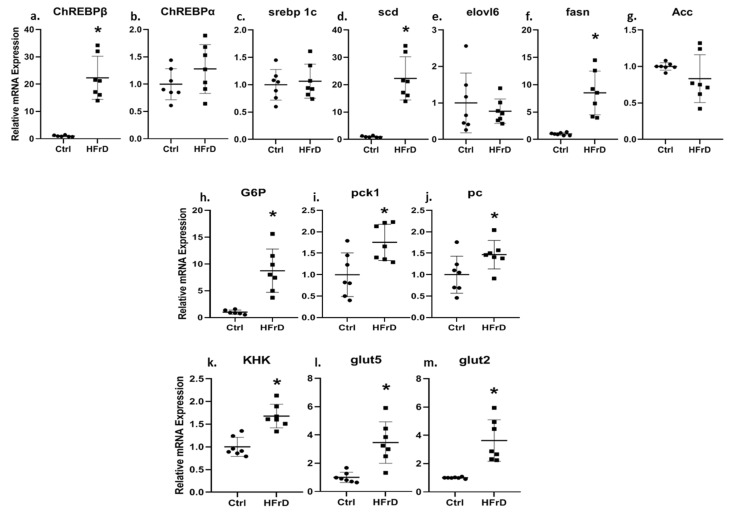
Fructose consumption increased *ChREBPβ* and its downstream genes RNA expression. HFrD increased the expression of genes in the kidney that regulate de novo lipogenesis (**a**–**g**), gluconeogenesis (**h**–**j**) and fructose intake and metabolism (**k**–**m**). Gene expression was measured by real time PCR. * *p* ≤ 0.05. *n* = 7–8.

HFrD increased the expression of the transcription factor *ChREBP*β 22-fold (Figure 3a). In contrast, no significant change was observed for *ChREBPα* (Figure 3b) and for the transcription factor srebp1c (Figure 3c). The scd and fasn genes, which are regulated by *ChREBP* and are part of the de novo lipogenesis (DNL) pathway, were upregulated significantly in HFrD-fed rats (Figure 3d,f). However, no difference was observed for the genes elovl6 and Acc, which are also regulated by *ChREBP* and are part of the DNL pathway (Figure 3e,g). Additionally, genes participating in the gluconeogenesis pathway such as G6P (which is directly regulated by *ChREBP*), *Pck1* and *Pc* were all upregulated by HFrD (Figure 3h–j). *KHK*, an enzyme that metabolizes fructose, and the two fructose transporters, *Glut5* and *Glut2*, were also upregulated in HFrD-fed rat kidneys (Figure 3k–m).

### 3.4. Fructose Increased the Expression of DNL Genes in Renal Cell Culture

**Figure 4:**
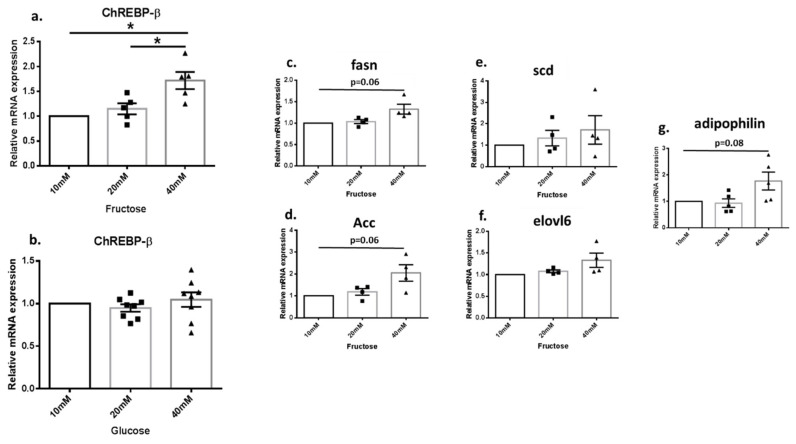
Fructose but not glucose upregulates *ChREBP*β expression in the human kidney cell line HK2. HK2 cells have been treated with fructose (**a**) or glucose (**b**), at 10, 20 and 40 mM, and *ChREBP*β expression level, were measured. Fructose-treated cells were also screened for the DNL *ChREBP*β downstream genes—*Fasn* (**c**), *Acc* (**d**), *Scd* (**e**), *Elovl6* (**f**) and adipophilin (**g**). * *p* ≤ 0.05. The results represent 4–8 experiments.

HK2 cells (a human kidney tubule cell line) were treated with 10, 20 and 40 mM glucose or fructose. In the fructose treated cells, *ChREBPβ* expression was upregulated in 40 mM comparted to 20 or 10 mM (Figure 4a). However, in the glucose-treated cells, the expression level of *ChREBPβ* was stable even in increasing glucose concentrations (Figure 4b). Subsequently, we looked for the DNL genes downstream to *ChREBPβ*. No significant changes were observed upon fructose treatment; however, the upregulation trend can be noticed for all the analyzed genes (Figure 4c–g).

## 4. Discussion

HFrD is linked to renal impairment. The mechanisms underlying this linkage have not been fully elucidated. Most studies attribute the kidney damage to fructose metabolites, including uric acid and methylglyoxal, and to oxidative stress (reviewed in [25]). In this study, we show increasing renal fat accumulation by HFrD, using a rat model for metabolic-like syndrome. In addition, fructose metabolism in the kidney was associated with an upregulation of *ChREBP*β and its downstream genes, *Scd* and *Fasn*, which participate in lipogenesis. Therefore, we conclude that HFrD, as part of its systemic metabolic effects, also induces “fatty kidney”, and that the origin of the ectopically accumulated lipids in the kidneys is de novo lipogenesis regulated by *ChREBP*β.

Previous studies have speculated about this hypothesis, and there is very little evidence that suggests that HFrD induced “fatty kidney”. De Castro et al. were the first to postulate that rats consuming a 60% fructose diet may develop renal ectopic fat accumulation. However, their findings were based on the presence of holes in the histological slices that are an indirect method for measuring fat accumulation [18]. There are two works that studied the effect of liquid fructose consumption. Fan et al. studied Sprague–Dawley rats that got 10% fructose in their drinking water. The study was designed to look for the effect of betaine inflammatory parameters caused by fructose. They showed increased Tg levels in serum and in kidney tissue of the rats, very similar to our results [26]. In 2020, Milutinovic et al. studied Wistar rats consuming 20% liquid high-fructose supplementation and showed renal de novo synthesized palmitate and the percent of palmitate in the total renal fat. However, no significant intra-renal lipid accumulation was observed [17].

The relevance of a fatty kidney to nephropathy development has been reported in a diabetic nephropathy mouse model, and in human patients with diabetes [27,28].

Andres-Hernando et al. reported that the genetic knockout of mouse fructokinase, the first enzymatic step in fructolysis, diminishes kidney injury in several acute kidney injury models [29]. In our study, the fructose transporters *Glut2* and *Glut5*, and *fructokinase* (KHK), were upregulate in HFrD, showing that chronic consumption of fructose may be absorbed by the kidney and cause renal chronic fructolysis, which may later contribute to CKD development.

We have shown that, consequently to fructose metabolism, *ChREBP*β is significantly upregulated. The damaging role of *ChREBP* upregulation in the kidney has been demonstrated in some animal models of diabetic nephropathy and chronic renal failure [27,30]. Kim et al. demonstrated that kidney *ChREBP* is upregulated in refeeding fasting mice [10]. Park et al. showed that treating mesangial cells with high carbohydrate (glucose) conditions results in *ChREBP* upregulation [31]. They also evaluated the role of *ChREBP* expression in kidney lipid accumulation and have shown that high glucose conditions result in intracellular lipid accumulation, whereas cells with mutant *ChREBP* exhibit less lipid accumulation [31]. Additionally, in DOCA-salt hypertensive rats, serelaxin treatment was shown to decrease lipid accumulation in the kidney in part by decreasing *ChREBP* (among other genes) [32]. Our study adds significant data, specifically regarding fructose metabolism and *ChREBP*. The previous studies did not distinguish between the different isoforms of *ChREBP*. However, from what is known about liver fructose metabolism, we separately tested for the two isoforms of *ChREBP* and found that only *ChREBP*β but not *ChREBP*α is upregulated in response to fructose consumption.

Genes participating in gluconeogenesis, including *G6P*, *Pck1* and *Pc*, were also upregulated in the kidney. These findings indicate that in addition to the liver, the kidneys also generate glucose in response to fructose consumption. Among these genes, G6P showed the most significant elevation. This gene encodes the glucose 6-phosphatase enzyme, which hydrolyzes glucose 6-phosphate to create a free glucose molecule that can be exported from the cell via glucose transporter membrane proteins. In the liver, G6P is regulated by *ChREBP*β, and an increase in hepatic G6P is sufficient to cause glucose intolerance and insulin resistance [22,33]. The role of G6P in the kidney has mostly been studied in the context of glycogen storage disease, where it is deficient [34,35]. The clinical implication of G6P upregulation in the kidney is still not clear and needs further studies. We believe that it can contribute to systemic insulin resistance as in the liver.

The impact of fructose on fat accumulation in the kidney can be direct or mediated by other organs. To see if there is a direct impact, we used a kidney cell line from a proximal tubule origin (the HK2 cell line) since it is the primary site for fructose metabolism in the kidney [16]. By treating these cells with fructose or glucose, we showed that fructose but not glucose directly upregulates *ChREBP*β expression. However, we did not see a significant upregulation in its downstream genes or in adipophilin, which mediate the DNL process.

The effect of HFrD on the kidney by intrarenal triglyceride accumulation, the upregulation of *ChREBP*β and its downstream genes, and the direct effect of fructose on kidney cells indicate that an HFrD affects kidneys by the same mechanism that it affects the liver, as described by Herman’s group [22].

One should notice that we presented only mRNA *ChREBP*β expression but not its protein expression. This is a limitation of the study; however, this limitation is derived from some objective difficulties in presenting this data. To date, there are no antibodies that can distinguish between the two gene isoforms. In addition, the half-life of the β isoform is very short, making it even harder to capture the protein itself.

## 5. Conclusions

Fructose studies normally focus on its effect on fatty liver induction through hepatic lipogenesis and gluconeogenesis. In our study, we have shown that identical pathways are also involved in kidney fructose metabolism. High-fructose consumption results in overexpression of the transcription factor *ChREBP*β and its downstream genes and leads to fatty kidney development.

## Figures and Tables

**Table 1:** A detailed comparison of the diets.

	Control (TD.170308) (g/Kg)	HFrD (TD.89247) (g/Kg)
Casein	207	207
DL-Methionine	3	3
Corn starch	400.15	---
Maltodextrin	200	---
Fructose	---	600
Lard	50	50
Cellulose	79.81	79.81
Mineralmixrogers-harper (170760)	50	50
Zinc carbonate	0.04	0.04
Vitamin mix Teklad (40060)	10	10
	% by weight %kcal from	% by weight %kcal from
Protein	18.3 21.4	18.3 20.2
Carbohydrate	55.4 64.9	60.4 66.8
fat	5.2 13.7	5.2 13
Kcal/g	3.4	3.6

**Table 2:** Primers, including GeneID, predicted amplicon size (bp), and abbreviations *.

Gene Name (GeneID)	Forward	Reverse	Predicted Amplicon Size (bp)
**Human**			
*Rplp0-B (6175)*	CCTTCTCCTTTGGGCTGGTCATCCA	CAGACACTGGCAACATTGCGGACAC	133
*ChREBPβ* (51085)	AGCGGATTCCAGGTGAGG	TTGTTCAGGCGGATCTTGTC	Primer design in [20]
*Scd* (6319)	GCAAACACCCAGCTGTCAAA	GCCAGGTTTGTAGTACCTCC	101
*elovl6* (79071)	AATGGATGCAGGAAAACTGGAAG	ACTGACTGCTTCAGGCCTTT	223
*Fasn* (2194)	CTTCAAGGAGCAAGGCGTGA	ACTGGTACAACGAGCGGATG	210
*Acc* (31)	AATCTTGAGGGCTAGGTCTTTT	GTCCAACTTCACCAGGTTGC	208
*Adipophilin* (123)	GCTGCAGTCCGTCGATTTCT	CCACACTCGGTTGTGGATCA	73
**Rat**			
*Rplp0-B* (64205)	GAACATCTCCCCCTTCTCCTTC	ATTGCGGACACCCTCTAGGAA	130
*Adipophilin* (25629)	GTCCATCTGATTGAATTCGCCA	CTCAGCACAATGGGACTCGT	135
*ChREBPβ* (171078)	TCTGCAGATCGCGCGGAG	CTTGTCCCGGCATAGCAAC	Primer design in [23]
*ChREBPα* (171078)	TGCATCGATCACAGGTCATT	AGGCTCAAGCATTCGAAGAG	163
*Srebp1c* (78968)	AGTTCCAGCATGGCTACCAC	GGGGTCTCTCAGTTTCCTGC	166
*Scd* (246074)	TGCTCTGGGGGATATTTTACTACC	GAGAAGAAAAAGCCACGGCG	238
*elovl6* (171402)	GAGGCGCAGAGAACACGTAG	CGCTTGTTCATCAGATGCCG	202
*Fasn* (50671)	AGCCTGAGCTTGTCCCTAGA	CACTGGTACACTTTCCCGCT	179
*Acc* (60581)	CTTGGGGTGATGCTCCCATT	GCTGGGCTTAAACCCCTCAT	116
*G6P* (25634)	CGTCACCTGTGAGACTGGAC	ACGACATTCAAGCACCGGAA	144
*Pck1* (362282)	GGATGTGGCCAGGATCGAAA	ATACATGGTGCGGCCTTTCA	172
*Pc* (25104)	CCAAGCAGGTTGGCTATGAGAA	GATGTTTTCCTGCCGCAGCC	206
*KHK* (25659)	ATGGCCATGTTGCCGACTT	TCTGGCAGGTTCGTGTCGTA	202
*Glut5* (65197)	CATGGTCACGGTTTTTGTGG	AGACGATGCTGACATAGGGC	149
*Glut2* (25351)	CGCACGCAACATGTCAGAAG	TTATTACCTCTTGAGGTGCATTGA	125

* *RPLP0*—Ribosomal Protein Lateral Stalk Subunit P0; *ChREBPα*—Carbohydrate Response Element-Binding Protein α; *ChREBPβ*—Carbohydrate Response Element-Binding Protein β; *srebp* 1c—Sterol Regulatory Element-Binding Protein 1c; *scd*—Stearoyl- CoA Desaturase; *elovl6*—Elongation of Very Long Chain Fatty Acids Protein 6; *fasn*—Fatty Acid Synthase; *Acc*—Acetyl-CoA Carboxylase 1; *G6P*—Glucose-6-Phosphatase; *pck1*—Phosphoenolpyruvate Carboxykinase 1; *Pc*—Pyruvate Carboxylase; *KHK*—Ketohexokinase; *Glut5*—Glucose Transporter Type 5; and *Glut2*—Glucose Transporter Type 2.

## Data Availability

The data presented in this study are available on request from the corresponding author.
